# "When I first saw a condom, I was frightened": A qualitative study of sexual behavior, love and life of young cross-border migrants in urban Chiang Mai, Thailand

**DOI:** 10.1371/journal.pone.0183255

**Published:** 2017-08-15

**Authors:** Arunrat Tangmunkongvorakul, Patou Masika Musumari, Kriengkrai Srithanaviboonchai, Veruree Manoyos, Teeranee Techasrivichien, S. Pilar Suguimoto, Masako Ono-Kihara, Masahiro Kihara, Suwat Chariyalertsak

**Affiliations:** 1 Research Institute for Health Sciences, Chiang Mai University, Chiang Mai, Thailand; 2 Department of Global Health and Socio-epidemiology, Kyoto University School of Public Health, Kyoto, Japan; 3 Japan Foundation for AIDS Prevention, Tokyo, Japan; 4 Faculty of Medicine, Chiang Mai University, Chiang Mai, Thailand; 5 Center of Medical Education, Graduate School of Medicine, Kyoto University, Kyoto, Japan; University of Toronto, CANADA

## Abstract

**Background:**

Many young migrant workers move across the border to Chiang Mai, a major city in Northern Thailand, in search of work opportunities. This study describes their sexual behavior, lifestyles, relationships and experiences with youth-friendly sexual and reproductive health (SRH) services.

**Methods:**

This is the qualitative arm of a mixed methods study using focus group discussions (FGDs) among young MWs aged 15–24 years in urban Chiang Mai. We conducted 6 FGDs with 84 participants (43 males, 41 females) organized in groups of 10–15 people, including 3 groups of males, 2 groups of females, and 1 group of both males and females.

**Results:**

We found that the lack of parental control, pressure to assimilate into Thai society, access to social media and modern communication technologies, and limited knowledge and access to sexual and reproductive health (SRH) services interplayed to shape lifestyle and sexual behaviors, including low condom use among young migrants.

**Conclusion:**

The present study helped discern the vulnerability of young migrants to adverse SRH outcomes. This particular group of youth needs urgent intervention to improve their knowledge on SRH and access to a youth-friendly clinic to help them personalize risk of HIV and other adverse SRH outcomes.

## Introduction

Labor migration has become one issue of particular salience for public health in Thailand. The country is home to approximately 3.25 million migrant workers (MWs); 2.7 million of whom are documented and undocumented MWs from Myanmar, Cambodia, and Laos [1]. This number is expected to increase given Thailand’s relatively higher economic status, which acts as a pull factor for MWs from neighboring countries.

Many studies in Thailand indicate that MWs are a particularly high-risk group for adverse sexual and reproductive health (SRH) outcomes such as HIV/STIs and unintended pregnancies. For example, studies have documented that this population has a high prevalence of HIV infection [2], low prevalence of condom use [3–5], high frequency of visits to sex workers [3, 4], and low prevalence of HIV testing [6]. The vulnerability of MWs to adverse SRH outcomes has been attributed to a range of factors, including poor social integration into the host country [7–9] and legal and financial barriers to services [7–11]. MWs without legal status may be reluctant to use available public health services due to fear of deportation. Similarly, income can be a barrier to accessing health services for MWs unaware of low or no cost health programs [7, 8]. There are a number of other factors, such as poor educational level and limited Thai language ability, that interplay to shape the vulnerability of MWs [12, 13].

In response to this, the Thai government, through the Ministry of Public Health, has taken a series of measures to improve access and quality of health care services for migrants. The main 4 activities include health examinations; necessary treatments; health promotion and disease prevention; and surveillance [14]. Other services outlined in the guidelines include health screening for high-risk groups (men who have sex with men (MSM), female sex workers, people who inject drugs, and cross-border MWs), family planning, disease control and prevention, and counseling for MWs [15]. Unfortunately, the implementation of these policies has not yet reached a national scale, and their effect on the health of migrants are yet to be seen. Moreover, these policies are not consistently reinforced and implemented, and do not cover undocumented MWs [16, 17].

Young MWs are a particular group whose sexual behavior patterns and lifestyle remain generally understudied. In 2012, the prevalence of HIV among MWs in Thailand was low, estimated at 0.8%, with young MWs carrying the highest burden. However, MWs displayed high levels of risk behaviors including multiple sexual partnerships and inconsistent use of condoms [18, 19]. In Thailand, young MWs accounted for approximately 20% of the documented 1,443,474 legal MWs in 2015. This proportion is similar for young MWs in Chiang Mai City, the setting of the current study, which hosts a total of 102,456 documented MWs [20]. Chiang Mai province is the economic epicenter of Northern Thailand; it is rapidly urbanizing, borders Myanmar and Lao PDR, and attracts a growing number of young MWs from these countries. These young people may have special SRH needs, which are yet to be thoroughly explored. Understanding their needs is therefore crucial for policy and program development.

The current study is the qualitative arm of a mixed-method study conducted to document HIV infection sexual risk behaviors among young cross-border MWs in Chiang Mai City. In our quantitative study [21], which included 442 participants aged 15–24 years, more than half (57%) of participants were sexually active, but the majority had never used condom, and a significantly low proportion of young MWs reported using condoms consistently. The majority of our participants were from the Shan ethnic group, [21] a group primarily from Shan State in Myanmar and constitutes one of the largest groups of cross-border MWs in Chiang Mai province [1, 22, 23]. The Shan in Thailand include two major groups. First are those who migrated to escape violence and human rights abuses due to political unrest and the armed conflict with Myanmar’s military junta. They are refugee migrants; many of whom are not officially recognized by the Thai Government. These workers are considered illegal undocumented workers. The second group includes both documented and undocumented Shan MWs who have migrated in search of economic opportunities in Thailand. Many Shan work in low-paid jobs including domestic work, construction, agricultural labor, and sex work [1, 22, 23]. Changes in social and traditional norms of the Shan living in Thailand have been reported anecdotally. These include high rates of divorce for groups in Northern Thailand [24]. However, little has been reported on the changes of lifestyle and sexual behavior of the Shan in Thailand. The few existing HIV-related studies that have included the Shan population have shown that this group of migrants was disproportionately affected with HIV, with prevalence rates much higher than in the general Thai population and other ethnic migrants from Myanmar [25–27].

This qualitative study specifically aimed to describe young migrants’ sexual and reproductive health behaviors, lifestyle, and awareness and use of youth-friendly services in urban Chiang Mai.

## Methodology

This qualitative study was conducted between March 2014 and February 2015. The target group was young cross-border MWs aged 15 to 24 years who lived in urban Chiang Mai. Participants in the qualitative study were recruited from among those who participated in the quantitative survey. A full description of the quantitative survey is described elsewhere [21]. The quantitative surveys were administered in areas where young migrants gathered, such as outdoor areas of residential camps, Buddhist temple, non-formal education centers for migrant workers, and various construction sites. Upon completing the quantitative survey, participants were informed of the focus group discussions (FGDs), and invited to participate. Recruitment for the qualitative study continued until a total sample size of 84 participants was reached. Participants interested in participating in the FGDs were provided with information regarding the objectives of the FGDs, and then were contacted by phone regarding the date of the FGD.

The focus group guide included open-ended questions specifically designed to probe young MWs’ about their daily lifestyle, intimate relationships and perceptions of SRH services in Chiang Mai (See S1 and S2 Files). The FGDs were conducted in rooms that ensured participants’ safety and privacy and participants were referred to using pseudonyms to conceal their identities. Participants were encouraged to freely answer the questions and share their opinions and experiences during the discussion. The FGDs were mainly conducted in Thai given that all the participants were relatively fluent. However, participants had the option of responding in their own languages (Shan and Burmese) if this was more comfortable for them. Our field research team consisted of 7 members, of whom three were fluent in Shan and Burmese and facilitated translation during the FGDs. Three research team members were present at each FGD, including one FGD moderator, two note takers (one fluent in Shan and Burmese). The FGDs lasted around 60–90 minutes.

### Ethics statement

The study received ethical approval from the Human Experimentation Committee, Research Institute for Health Sciences, Chiang Mai University (Certificate of Ethical Clearance No. 52/2514). All participants gave verbal informed consent or assent, and written consent (signature or fingerprint) from a guardian was received for participants aged 15–17 years old. The locations for focus group discussions were selected to maximize participants’ sense of safety and comfort. The FGDs were conducted at the places where participants felt safe and comfortable to provide information, such as the quiet places in non-formal education centers for MWs, young migrant Learning Center Building, and outdoor areas at play grounds or living camps where participants used for meeting friends, playing sports, etc. We used pseudonyms to preserve confidentiality of participants during the FGDs.

### Data analysis

The FGDs were digitally audio recorded, transcribed verbatim, and translated into Thai (for responses that were in Shan or Burmese). The transcripts were annotated with field notes taken during the interviews. The transcripts were coded and analyzed using content analysis. The process included identification of repeated normative themes, which emerged either spontaneously from the discussion or directly from the responses to the open-ended questions designed for the study. Passages most relevant to the study were later translated into English for presentation in manuscripts.

## Results

A total of 84 participants, including 43 males and 41females, were recruited. We conducted 6 FGDs of 10–15 participants, specifically including 3 groups of males, 2 groups of females, and 1 group of both males and females. All the participants were Shan by ethnicity.

The themes that emerged from the FGDs are presented below, and are supported by quotes from participants. In some instances, the quotes were lightly edited to facilitate reading and understanding, but without altering the original meaning.

### Contemporary lifestyles and sexual relationships

#### Lack of parental control and sense of freedom

Most participants reported that the lifestyle they currently lead in terms of love, relationships, and sexual behavior in Thailand do not reflect how they would behave in their home country. The traditional values of Shan culture, and by extension of their families, discourage premarital sex, teenage romantic relationships, and cohabitation of unmarried couples. Participants reported, for example, that it was considered immoral for an unmarried female to go on a date with a man or to be touched by a man. They explained that their parents closely monitored them with regards to relationships and dating while still in Myanmar before they migrated to Thailand.

Young migrants felt that being away from their parents offered them an opportunity to express their sexuality and to love more openly without having to deal with the consequences of disobeying and disappointing their parents. They found it easier to associate with other young migrants with whom they shared common new living conditions.

“*I think they [young migrants] are at risk because they are staying with friends*. *If they have friends encouraging them to drink*, *to go out at night or to visit boys*, *it is very easy for them to do so because there are no adults to stop them*.”

(Young female migrant, female focus group)

Many participants had a sense a freedom living in Thailand as they could get together with friends more freely than they did when they were in Myanmar. Festivals and other important local and public holidays in Thailand were an opportunity for young migrants to meet with peers and to seek out potential sexual encounters.

“*It is different here than at home [in Myanmar]*, *because at home*, *I didn’t have the chance to go out that much*. *But here it is like I live in a hotel*, *I have everything in my place [in Thailand] that my house [in Myanmar] doesn’t have*. *I can go out anywhere I want*.*”*

(Young female migrant, female focus group discussion)

#### Parental monitoring from home countries

Other participants reported that their parents worried about their promiscuity and repeatedly warned them to be careful in their relationships. Their parents encouraged them to follow the traditional rules for love and marriage if they were in a relationship, and warned them not to engage in inappropriate behaviors such as premarital sex.

“*Sometimes my parents are worried about me and when they are worried*, *they call me more often*. *Some people back home told my parents that being in Thailand puts one at risk of getting AIDS or having an unintended pregnancy*, *and that Shan girls in Thailand wore short skirts that were not appropriate*. *This makes my parents worry a lot*.”

(Young female migrant, female focus group discussion)

“*When I go out with a boy*, *I worry because my parents would tell me to marry that boy*. *They would tell me not to go out with him if I didn’t really love him because [casual dating] is not proper behavior for good girls*.”

(Young female migrant, female focus group discussion)

#### Adoption of the local lifestyle

Many young MWs reported that their lifestyle in Thailand is different than their lifestyle in Myanmar. This included how they dressed and spoke, or their approach to dating and sexual relationships.

“*Here there are so many beautiful Shan women and they are tempting*. *When I was back home*, *young women dressed modestly and wore traditional skirts*, *but they don’t do that here in Thailand*.”

(Young male migrant, male focus group discussion)

“*Right now my friends more closely resemble Thai people*, *unlike my friends in Myanmar*. *Their outfits*, *thoughts*, *and even their vocabulary is more like Thai people*. *They don’t speak Shan anymore*. *When they meet at a Shan temple fair [in Chiang Mai]*, *they speak Shan and wear Shan outfits*, *but when they leave [the fair] and go to work*, *they don’t really speak Shan*.*”*

(Young male migrant, male focus group discussion)

#### Access to social media and modern communication technologies

Access to the Internet, mobile phone technologies and social networking sites emerged as an important factor that impacted young MWs’ lifestyle, dating, and relationships. Many reported having greater access to modern forms of communication (e.g. mobile phones, smartphones and tablet computers) in Thailand. This made it easy for them to reach out and communicate with one another, and to initiate new romantic relationships through social media.

“*Nowadays*, *it is easier to have a relationship because of social media—the Internet allows us to chat*, *to see each other and go out together*. *It is much more convenient compared to the past back home*.*”*

(Young male migrant, male focus group discussion)

“*Back home [Myanmar] it is not easy to have a relationship with a woman*. *But in Thailand*, *if we can get her phone number*, *we can chat via Line which means that we can have a relationship*.”

(Young male migrant, male focus group discussion)

### Knowledge, attitudes, and experience using condoms and contraception

#### Poor knowledge of and use of condom

Although some participants, particularly males, had positive attitudes that young people should carry condoms, and reported using condoms for preventing both STIs and unintended pregnancies, many of the participants in this study still had limited knowledge regarding how to appropriately use a condom. Most participants who stated not knowing how to use a condom had never seen one before. Additionally, male participants felt that young female migrants, compared to them, had even worse knowledge about condoms.

“*I think many Shan women don’t know about condoms*. *Some women may even ask what it is*. *Even if they see it*, *they don’t know how to use it*. *Actually*, *some men are worried that women will see the condom if they carry one around*. *But back home [in Myanmar] they don’t need to worry about this because no one knows what it is*.”

(Young male migrant, male and female group discussion)

On the other hand, female participants were shy about talking about issues regarding condoms. They acted embarrassed when shown a condom, and were scared to touch the package (when it was presented at the end of the FGD).

There was also a lot of stigma and taboo around issues related to condoms.

“*When I first saw it [condom]*, *I was frightened*, *I had never seen it before*.. *I brought it [given by a non-government organization (NGOs)] to my boss and asked him what it was*. *I didn’t know*. *He told me that it was something for males to use*.”

(Young female migrant, female group discussion)

“*One day*, *while I was in class*, *a teacher told us about condom*., *I then visited my relatives showed them the condom*. *My relatives thought that it was something dirty to talk about*. *That day*, *I was scolded badly (laughs)*. *… For the Shan*, *this is an unacceptable issue to talk about*. *If we talk about condoms*, *no one dares to express their opinions*.”

(Young female migrant, male and female focus group discussion)

#### Relationship trust and condom use

We found that condom use during sexual intercourse depended on the type of relationship and trust. Some male participants said that they would prefer using contraceptive pills over condoms, especially if they trusted their female partner. They based their trust of their female partner through observation of their behavior and by asking friends.

“*I rarely use condoms even though I see her [regular partner] once in a while*. *I prefer my girlfriend to take birth control pills*. *I don’t like to wear condoms*.”

(Young male migrant, male focus group discussion)

“*I will tell you frankly I have never used a condom once in my life and I have sex only with my girlfriend*. *I don’t know how to use it*. *I just learned about it today [from a focus group discussion] (sound of laughing from male participants)*. *She uses the pill*.”

(Young male migrants, male focus group discussion)

#### Poor knowledge and use of contraception

We found that there were gender differences regarding knowledge of contraception. Male participants knew many more contraceptive methods than their female counterparts whose knowledge of contraception was mostly limited to contraceptive pills. Some young female MWs expressed worries about the side effects of other methods of contraception such as contraceptive implants and were reluctant to use them. They feared that some methods could potentially affect their ability to get pregnant.

“*I have never seen injection birth control*. *The inserting medicine one [contraceptive implant] seems dangerous*. *It scares me*.”

(Young female migrant, female focus group discussion)

“*I know a person back home [in Myanmar]–who after receiving a birth control shot*, *things were no longer normal*. *She didn’t know when her period would come*. *But with birth control pills*, *we know that the period will come right after we finish the pills*, *and after the period is finished*, *we can start to take the pills again*. *It is easy like that*.”

(Young female migrant, female focus group discussion)

### Use of youth friendly health services

#### Access to service: Unawareness of the eligibility right

Most participants were neither aware of the existence of youth-friendly clinics nor of their eligibility to access the services under the MW health insurance system.

“*I wake up in the morning*, *go to work and go back to my place*. *I don’t go out that much*, *so I don’t know*. *None of my friends talk about this clinic*. *I don’t have any information about that kind of service for the young*.”

(Young male migrant, male focus group discussion)

“*I have never heard of a service like that for young people*. *Is there really one*?*”*

(Young female migrant, female focus group discussion)

#### Need for tailored sexual and reproductive health services for young migrants

The need for tailored SRH services did not spontaneously emerge from the FGDs. We did however ask participants what kind of services they would want to receive if youth-friendly clinics were available. Most indicated that youth-friendly services should ideally be provided in their native language and should include programs to improve knowledge about SRH. They expected such programs to provide them with the right knowledge and skills, including condom use, contraception, STIs and HIV/AIDS, so that they can make informed decisions and adopt appropriate behavior in times of need.

“*If it is possible*, *I want Shan people who have knowledge on this thing [sexual and reproductive health issue] to be the health providers or trainers*. *Because most people here [young migrants] are Shan*, *we will communicate well in our own language*.*”*

(Young male migrant, male and female focus group discussion)

## Discussion

The findings from our qualitative study corroborate results from our quantitative survey [21], and expand our understanding of the factors that shape sexual and reproductive health behaviors among young MWs in Chiang Mai. What was alarming from the results of our quantitative survey was that a high proportion of sexually active MWs never used condoms, and very few used condoms in a systematic fashion [21]. In our qualitative study, we found that there is an interplay of factors that can be useful in discerning the vulnerability of MWs to HIV and adverse SRH outcomes. For example, many participants in our study reported not knowing how to use a condom, and some, particularly females, had never seen a condom prior to the FGDs. There was a lot of social stigma and taboo around issues related to condoms, predominantly among females. Additionally, the type of relationship and trust influenced condom use. Quite a few male participants found that the use of condoms was not justified in a committed romantic relationship where trust prevails, and they preferred the use of contraceptive pills to condoms. These factors can partly explain the low prevalence of condom use in our quantitative survey since most (91.7%) had initiated sexual activity with their girlfriend or boyfriend, rather than with a casual or a commercial sex worker [21]. The evaluation of the PHAMIT-2 project (Prevention of HIV among Migrant Workers in Thailand), which compared baseline (2010) [11] and end line (2014) [28] surveys, found that the prevalence of “ever used a condom with a regular partner or spouse” remained very low despite the modest changes observed over the two rounds of surveys, from 11% to 19%, while the proportion of MWs reporting “never used a condom with a non-regular partner in the past 12 months” declined from 42% to 21% [28]. There is an urgent need to develop strategies to help MWs personalize the risk of HIV/STIs and unintended pregnancies regardless of the type of sexual partner.

Anecdotal reports suggest that the migration of Shans to Thailand has led to a change in social norms and to the traditional Shan community; citing for example the extremely high rates of divorce, averaging 90% in some areas of Northern Thailand [24]. However, very little is known about how these changes in social and traditional norms impact the sexual behavior of young MWs. The results of our quantitative survey indicated that most MWs had initiated sexual activity at an early age and had resided in Thailand for less than 5 years, suggesting that they might have been already sexually active before migrating to Thailand [21]. In our study, young MWs attributed the changes in their life style and sexual behavior to the lack of parental monitoring. They also linked access to social networking and modern communication technologies to their ability to initiate new romantic and/or sexual relationships. There has been an unprecedented growth in social network technologies over the past decade. A number of studies from both developed and developing settings have associated the use of these technologies with increased risky sexual behaviors [29–31]. In a recent study from Swaziland [29], participants who found it easier to initiate a romantic conversation on Facebook were more likely to report multiple sexual partnerships. The explosion of social networking sites, particularly among the young –including young migrants-, makes it an attractive channel to reach out to this vulnerable group. Future research should thoroughly explore the patterns of use of social media and other communication technologies among young MWs, and the feasibility to use them as potential channels for health interventions.

It is very concerning to find that young MWs were neither aware of the availability of youth friendly health services, nor of their eligibility to access the services under the MW health insurance scheme. The participants expressed a need for such services, and indicated that it should ideally be provided in their native languages and be tailored to their needs. It is worth noting that the participants’ need for youth-friendly SRH services did not emerge spontaneously; rather, it was expressed in response to a specific question asking them what kind of services they would want to receive if youth-friendly SRH services were available. In view of our quantitative survey which showed that a low proportion (41.2%) had health insurance [21], interventions to improve access to youth-friendly health services in Thailand should, at the individual level, aim to increase young MWs’ awareness of the availability of youth-friendly services as well as their eligibility for these services, and at the structural level, to increase health insurance coverage for the migrant population.

Thailand has been successful in mounting programs and policies that have rapidly led to the improvement SRH services in the country. Most programs and policies however had targeted married women, leaving other groups, such as adolescents and youths and MWs, with unmet needs for SRH services [32]. The nationwide establishment of youth-friendly SRH services is one of the initiatives the Thai Ministry of Public Health spearheaded to address the gap in SRH services among young people [33]. However, there are still many challenges to making the services acceptable by young people [33, 34].

It is anticipated that the provision youth-friendly SRH services to young migrants may present even more challenges than to the Thai population. For example, undocumented migrants and those without work permits, respectively 14.7% and 26.2% in our survey [21], may be reluctant to engage with the health or legal system for fear of being deported back their home countries. Another challenge is the government’s regulation to control the inflow, living, and spatial mobility of MWs in Thailand, which affects migrants’ access to health services [35]. Lastly, the unfamiliarity of young migrants with the Thai law, regulations, and health systems may create fear and impede access to SRH services even when they are entitled to receive such services.

This study has many limitations worth being mentioned. Firstly, we did not address issues of sexual orientation, including the types of sexual intercourse (male-to-male vs. male-to-female, anal vs. vaginal, etc.). The prevalence and incidence of HIV infection is very high in young MSM in Thailand, with orders of magnitude reaching eight-fold greater than in heterosexual men [36–38]. Similarly, we did not collect information regarding involvement in sex work. Migrant male sex workers (MSWs) are an extremely vulnerable population. In Chiang Mai, the majority of migrants MSWs are Shan from Myanmar. Many have unstable employment; have experienced sexual abuse, and often use recreational drugs [39, 40]. The lack of information on sexual orientation and involvement in sex work limits the interpretation of risk in our sample of participants. Future research will do well by exploring condom use and other sexual behaviors of MWs in light of their sexual orientation as well as involvement in sex work. Secondly, there is the possibility of social desirability bias in our study given the vulnerability of the young MWs and the use of a group format to discuss sensitive sexual issues. Our participants may have felt uncomfortable about describing their sexual practices and relationships. This can possibly explain why issues such as the sexual orientation of participants did not spontaneously emerge from the discussions. Social desirability bias can to a certain extent explain the apparent lack of knowledge about condoms among Shan female participants. In settings where gender double standards in sexual norms still prevails, such as in many Southeast Asian countries including Thailand and Myanmar [41, 42], females may prefer to be silent regarding their knowledge of sexual issues including condoms in an open forum due to fear of being regarded as sexually experienced; hence, being labeled as a “bad girl”. Lastly, our study did not explore in-depth the issue of contraception in light of the sexual culture of young migrants. We sought to understand the level of knowledge about existing contraceptive methods; however, delving deeper in the sexual culture of young MWs could have provided a deeper understanding of the use of contraception in this population.

Despite these limitations, this study provides a useful qualitative assessment of young MWs’ sexual behavior, lifestyles and relationships, and issues related to access to youth friendly health services in Chiang Mai, Thailand. Based on the findings from our study, as well as results from previous research [21, 28, 43–45], we suggest that health interventions for young MWs should be holistic, designed based on relevant contextual factors (e.g. emphasizing condom use regardless of the type of partner), using relevant channels of communication (social network sites, and other ICT technologies, etc.), address a range of outcomes (risky sexual behaviors, HIV testing, etc.), and target different levels of influence (individual, community, and structural: policy and regulations). This will require tight collaboration between all the interested stakeholders particularly the Ministry of Public Health, Provincial Health Office, Provincial Employment Office, hospitals and non-government organizations.

## Conclusion

The present study helped discern the vulnerability of young migrants to adverse SRH outcomes. We have highlighted a set of factors namely poor knowledge and lack of awareness of condoms, contraception, and SRH services that interplayed to shape the SRH behavior such as low condom use. The use of youth-friendly SRH services was limited by the lack of awareness of the existence of such services. Ideally, interventions to address SRH needs should not only improve knowledge and raise awareness on SRH issues, but also address upstream structural barriers to accessing health services such as lack of health insurance.

## Supporting information

S1 File Focus group discussion guide, Thai version. (DOCX)

S2 File Focus group discussion guide, English version. (DOCX)
