# Supplementary material for: "When I first saw a condom, I was frightened": A qualitative study of sexual behavior, love and life of young cross-border migrants in urban Chiang Mai, Thailand
Source: PLoS One. 2017 Aug 15;12(8):e0183255. doi: 10.1371/journal.pone.0183255 (PMC5557483; doi:10.1371/journal.pone.0183255)
Supplement: S1 File — (DOCX) [file pone.0183255.s001.docx]

**แนวคำถามสำหรับการสนทนกลุ่ม**

**(สำหรับกลุ่มเยาวชนแรงงานข้ามชาติ)**

**ข้อมูลด้านสังคมและเศรษฐกิจ**

- ท่านอายุเท่าไหร่?
- ท่านเกิดที่ไหน? ขณะนี้ท่านอาศัยอยู่กับใครอย่างไรบ้าง?
- ท่านช่วยกรุณาบอกข้อมูลเกี่ยวกับครอบครัวของท่านได้หรือไม่?
- ท่านไปโรงเรียนหรือเปล่า? ช่วยเล่าเกี่ยวกับการทำงานของท่านให้ฟังได้ไหมว่าทำหรือไม่หรือทำอะไร?

**วิถีชีวิต**

- ท่านใช้ชีวิตอย่างไรบ้างในระหว่างวันธรรมดาและวันหยุด?
- ท่านใช้เวลาว่างทำอะไรบ้าง?
- ท่านใช้เวลาอยู่กับเพื่อนหรือไม่อย่างไร? ช่วยเล่าให้ฟังหน่อยได้ไหมว่าเพื่อเป็นใครและทำอะไรด้วยกันบ้าง?
- ท่านใช้จ่ายเงินอย่างไรบ้างในชีวิตประจำวัน? ใช้จ่ายเงินในเรื่องใด? ท่านหาเงินมาเองหรือไม่อย่างไร?
- ท่านรับฟังและรับรู้ข้อมูลข่าวสารจากแหล่งข้อมูลหรือสื่ออะไรบ้าง?
- ท่านใช้คอมพิวเตอร์ /อินเตอร์เน็ต / โทรศัพท์มือถือ ในชีวิตประจำวันมากน้อยแค่ไหน? ท่านใช้มันอย่างไรหรือเพื่ออะไรบ้าง?
- ท่านดื่มสุรา สูบบุหรี่ หรือเสพสารเสพติดอื่นใดหรือไม่อย่างไรบ้าง? ลองเล่าให้ฟังหน่อยได้ไหม?

**ความรักและความสัมพันธ์**

- ตามความคิดเห็นของท่านการมีความรักความสัมพันธ์ของคนหนุ่มสาวในชุมชน/สังคมของท่านเป็นอย่างไร?
- เพื่อน ๆ ในสังคมของท่านมีความสัมพันธ์กับแฟนอย่างไรกันบ้าง?
- เขาทำอะไรกันบ้างตอนไปออกเดท?
- ตัวท่าน/วัยรุ่นคาดหวังอะไรกันบ้างสำหรับการออกเดท?

**การส่งเสริมให้มีเพศสัมพันธ์ที่ปลอดภัย**

- ท่านมีความคิดเห็นและความรู้สึกอย่างไรบ้างเกี่ยวกับถุงยางอนามัยกับวัยรุ่น?
- ท่านมีความคิดเห็นเกี่ยวกับการคุมกำเนิดอย่างไรบ้าง?
- อะไรบ้างที่เป็นอุปสรรคในการเข้าถึง/การใช้ถุงยางอนามัยและการคุมกำเนิดของวัยรุ่นในสังคมของท่าน?
- ท่านเคยได้ยินเกี่ยวกับคลินิก/บริการที่เป็นมิตรสำหรับวัยรุ่นไหม?
  - ถ้าเคย ท่านเคยไปใช้บริการไหม? มันเป็นอย่างไร?
  - ถ้าไม่เคย ท่านทราบหรือเปล่าว่ามันเป็นอย่างไร? ท่านสนใจที่จะไปใช้บริการหรือไม่?
- ถ้ามีคลินิก/บริการที่เป็นมิตรสำหรับวัยรุ่นสำหรับเยาวชนในสังคมของท่าน (กลุ่มแรงงานข้ามชาติ) ท่านคิดว่าการให้บริการควรจะเป็นอย่างไร?
