# Supplementary material for: "When I first saw a condom, I was frightened": A qualitative study of sexual behavior, love and life of young cross-border migrants in urban Chiang Mai, Thailand
Source: PLoS One. 2017 Aug 15;12(8):e0183255. doi: 10.1371/journal.pone.0183255 (PMC5557483; doi:10.1371/journal.pone.0183255)
Supplement: S2 File — (DOCX) [file pone.0183255.s002.docx]

**Focus group discussion guidelines**

**(For young migrant worker group)**

**Socio-demographic background**

- How old are you?
- Where were you born? What is your living arrangement now?
- Can you tell me a little about your family?
- Do you currently go to school? Can you tell me about your work / paid activity?

**Lifestyles**

- How do you spend your time during weekdays, weekends or holidays?
- What do you do during your free time?
- Do you spend time with friends? Can you tell me more about who your friends are and what you do together?
- How much money do you typically have to spend in your daily life? Where do you get it from? What do you spend it on?
- What types of mass media do you come into contact with?
- Do you have access to computer / Internet / mobile phone? What purposes do you use it/them for?
- Have you had experiences of drinking alcohol, smoking tobacco or using drugs? Can you tell me more about this?

**Love and Relationships**

- In your opinion, what are the love and relationships between boys and girls in your community like?
- How do your friends in your community have the relationships with their loved ones?
- What do they do on dating?
- What have you/young people expected from your/their dating relationships?

**Sexual health promotion**

- What is your opinion about condom use and young people?
- What do you think about contraception / birth control?

1. What are the obstacles to use/access to condom or contraception among young people in your community?

- Have you ever heard about a youth friendly clinic/youth friendly service?
  - If yes, have you ever used the service? How was it?
  - If no, do you know what it is like? Are you interested to use the service?
- If there is a youth friendly clinic/service for young people in your community (young migrants), what do you think the service should provide?
